# Uncemented custom femoral components in hip arthroplasty: A prospective clinical study of 191 hips followed for at least 7 years

**DOI:** 10.3109/17453674.2010.501748

**Published:** 2010-07-16

**Authors:** Pål Benum, Arild Aamodt

**Affiliations:** Department of Orthopaedics, St. Olav's University Hospital and Department of Neuroscience, Norwegian University of Science and Technology, TrondheimNorway

## Abstract

**Background and purpose:**

We have developed an individually designed, uncemented femoral component for achievement of improved strain distribution and fixation to the bone, to make uncemented stems more applicable in femurs of abnormal size and shape, and to improve the joint mechanics. Here we describe the design of the implant and present the results of a prospective clinical study with at least 7 years of follow-up.

**Patients and methods:**

The prostheses are produced by CAD-CAM technique. The design of the stem is based on CT information, and the neck design is based on the surgeon's planning of the center of rotation, femoral head offset, and leg length correction. The first-generation stem produced before 2001 had a proximal HA coating and a sand-blasted distal part that was down-scaled to avoid contact with compact bone. The second-generation stem had a porous coating beneath the HA layer and the distal part of the stem was polished.

The implant was used in 762 hips (614 patients) from 1995 until 2009. 191 of these hips were followed for 7 years and 83 others were followed for 10 years, and these hips are included in the present study. Mean age at surgery was 48 (20–65) years. Congenital dysplasia of the hip was the reason for osteoarthritis in 46% and 57% of the hips in respective groups. Merle d'Aubigné score was recorded in 152 and 75 hips in the two groups. Prostheses followed for 10 years, and almost all in the 7-year group, were first-generation stems.

**Results:**

The 7- and 10-year cumulative revision rates were 1.1% and 2.4%, respectively, with stem revision for any reason as endpoint. The clinical results were similar at 7 and 10 years, with Merle d'Aubigné scores of 17. Intraoperative trochanteric fissures occurred in 2 of the 191 operations (1.0%); both healed after wiring. In hips followed for 7 years, 2 periprosthetic fractures occurred; exchange of the stem was necessary in both. One additional fracture occurred between 7 and 10 years, and it was treated successfully with osteosynthesis. The rate of dislocation was 1.6% and 2.4%, respectively. There was no radiographic loosening at follow-up.

**Interpretation:**

Use of a custom femoral stem gives a reliable fixation and promising medium-term clinical results in femurs of normal and abnormal shape and dimension. The individual design, which enables optimized joint mechanics, gives a low risk of mechanical complications.

## Introduction

Several research groups have been involved in the development of uncemented customized femoral stems to optimize the fit of the stem to the femur and to optimize the joint mechanics (Robertson et al. 1987, Aldinger et al. 1988, Bargar 1989, Mulier et al. 1989, Stuhlberg et al. 1989, Rubin et al. 1992).

In 1990, a research group at our institution was established with the aim of developing a custom femoral stem. Using individual design, we aimed to improve the strain distribution and fixation to the bone in uncemented stems. We felt a particular need for individually shaped stems for use in hips with abnormal size and shape of the proximal femur. During the following 5 years, several experimental studies were performed to develop customized femoral stems with optimal fit to the proximal femur based on CT information and CAD-CAM technology. Furthermore, studies were carried out to document the stability of the stem and also the strain shielding in the proximal femur following insertion of the stem in human cadaver femurs (Kvistad et al. 1994, Aamodt et al. 1999, 2001, 2002). The custom femoral stem that was developed has now been in clinical use since 1995.

The aim of this paper is to present the design process of the custom femoral stem. We also report the 7–10-year clinical results using this implant.

## Patients and methods

### Surgical planning

**Figure 1.**
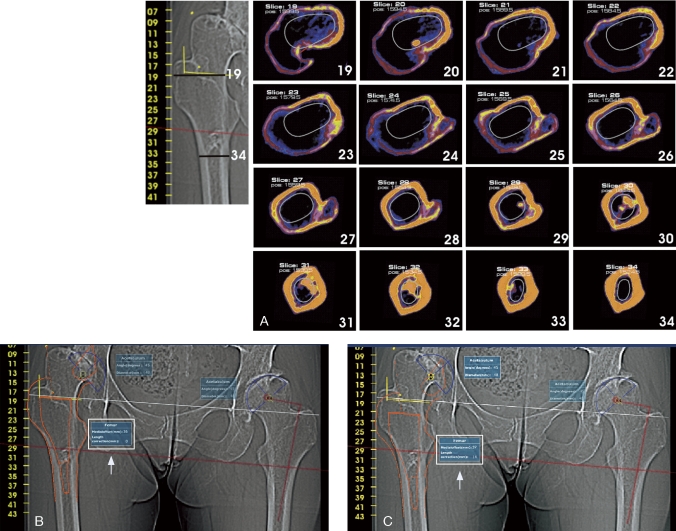
A. CT scans of the proximal femur, with 5-mm distance between each section. B. Digital template of the suggested stem design. C. Use of the template for determination of ideal medial femoral head offset and leg length. Information about medial femoral offset and leg length correction is automatically shown during use of the template. (The arrows show the text box where this information is given).

A CT scanning of the proximal femur and the pelvis is performed according to a standard protocol. The distal part of the femur is included to determine the femoral neck anteversion. The digitized CT information is sent to the manufacturer (Scandinavian Customized Prostheses (SCP), Trondheim, Norway). The surgeon has to provide a special order form along with clinical information, using a web-based interface to communicate with the manufacturer. The manufacturer then performs computer-aided modeling of the stem, aiming at contact with compact bone proximal to a level that is approximately 2 cm below the lesser trochanter and with a gradually increased offset between the stem and the bone distal to that level. Compact bone has been defined as bone with a CT density of more than 600 HU (Aamodt et al. 1999). A scanogram of the pelvis and the proximal femur and also a digital template of the suggested stem design are sent to the surgeon over the internet. The surgeon has to approve the suggested contours of the stem. Furthermore, the surgeon determines the center of rotation to achieve adequate medial offset and leg length by use of a digital template on the scanogram (Figure 1). The prosthesis is designed with a neck that gives a femoral neck anteversion of 10 degrees after insertion, unless the surgeon decides otherwise. Finally, the prosthesis (Unique; SCP, Trondheim, Norway) is manufactured using 3-axis CNC-machining. Stems designed before January 2001 were grit-blasted, and the proximal part of the stem was covered with HA only (CAM Implants, Leiden, the Netherlands) and sterilized by gamma technique (Gamma-Master BV, the Netherlands). Since we experienced that the stems of this first generation were slightly oversized, which necessitated removal of much compact bone, particularly in wide femurs with thick cortical bone, the stems were downscaled slightly from January 2001. The surface treatment of the second-generation stems was also changed: the proximal part of the stem was covered with a porous layer on which a 50-μm HA layer was added, and the stem distal to the coated area was highly polished (Medical Group, France).

### The operation

**Figure 2.**
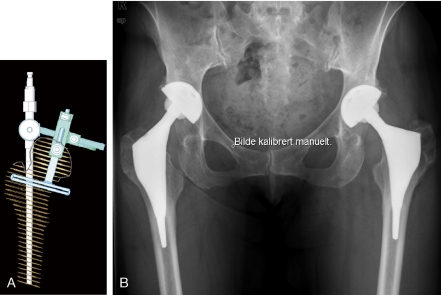
A. Adjustable resection guide. B. The prosthesis produced after the planning shown in Figure 1 has been inserted into the right femur. A custom prosthesis has also been implanted into the left femur.

All operations were performed in the lateral decubitus position with direct lateral approach. The planning documents from the manufacturer include information about the resection level of the femoral neck. Using a resection guide (Figure 2), the surgeon is able to reproduce the computer-planned resection accurately. The acetabular component chosen is then inserted following preparation of the acetabular cavity. An inclination of 45 degrees and anteversion of 10–20 degrees are intended. After preparing the femur with standard broaches according to the instructions from the manufacturer, the final preparation of the proximal femur is performed with 1 or 2 custom broaches. In cases with very compact bone, a broach that is somewhat smaller than the final one is used. A trial neck and head is then mounted on the final broach to determine an adequate neck length. The broach is then replaced with the custom femoral stem and the joint is reduced following mounting of the chosen prosthetic head.

The acetabular component and type of articulation are chosen according to the preferences of the surgeon. Prophylactic antibiotic with first-generation cephalosporin was given intravenously before surgery and at 8, 16, and 24 h postoperatively. LMWH was given subcutaneously for 2 weeks as thrombosis prophylaxis.

### Patients and prosthetic components

762 custom prostheses had been implanted by December 31, 2008. Of these, 191 had been followed for 7 years and 83 for 10 years (Table 1). 60% of the custom stems were used in women. Mean age at operation was 48 (20–65) years. Primary osteoarthritis was seen in 30% of the hips; 46% of the hips were dysplastic, whereas various other disorders were the reason for development of osteoarthritis in the remaining 24%. The percentage of primary osteoarthritis was considerably lower in hips observed for 10 years than in hips observed for 7 years (Table 1). Abnormalities in the shape of the femur such as abnormal femoral neck anteversion were more frequent in the group with 10 years of follow-up than in the group with only 7 years of follow-up (Table 2).

**Table 1.** Study samples

	7 years of follow-up (191 hips)		10 years of follow-up (83 hips)	
	n	%	n	%
Mean age at operation (years)	48 (20–65)		46 (20–60)	
Females	115	60	47	57
Males	76	39	36	44
Primary osteoarthritis	58	30	16	19
Hip dysplasia	88	46	47	57
Legg-Calvé-Perthes disease	14	7	10	12
Rheumatoid disease	16	8	5	6
Sequel. femoral neck fracture	3	2	1	1
Avascular femoral head necrosis	7	4	2	2
Other disorders of the hip	5	3	2	2

**Table 2.** Preoperative femoral neck anteversion

	7 years of follow-up (191 hips)	10 years of follow-up (83 hips)
Preoperative neck anteversion		
mean	19.5°	20.7°
range	-6° to 90°	-33° to 90°
Number of hips with		
retroversion	8 (4%)	3 (4%)
30° or more anteversion	40 (21%)	25 (30%)
50° or more anteversion	9 (5%)	6 (7%)

The types of custom stems, femoral heads, and acetabular components are shown in Table 3. All hips of the 7- and 10-year follow-up groups received a custom stem of the first generation except for 8 of the 7-year group. The majority of acetabular components were uncemented whereas most of the modular heads were ceramic.

**Table 3.** Acetabular and femoral components

	7 years of follow-up (191 hips)		10 years of follow-up (83 hips)	
n	%	n	%
Femoral components				
First generation	182	95	83	100
Second generation	8	4	0	0
Femoral heads				
Articul/eze	67	35	55	66
Ziolox	65	34	26	31
Biolox forte	59	31	2	2
Acetabular components				
Duraloc	173	91	76	92
Charnley	6	3	3	4
Elite Plus Ogee	7	4	4	5
Trilogy AB	5	3	0	0

### Preoperative protocol, postoperative follow-up

The preoperative protocol included clinical evaluation using the modified Merle-d'Aubigné score (d'Aubigné and Postel 1954) in addition to the radiological investigation of the pelvis and both hips and the CT investigation.

The initial protocol included clinical and radiographic follow-up 3 months, 6 months, 1 year, and 2, 3, 5, 7, 10, 13, 15, and 20 years postoperatively. Following 5 years' experience with the prosthesis, the protocol was modified to exclude the follow-ups at 6 months and at 2 and 3 years. We also excluded a regular recording of the Merle d'Aubigné score at the 7-year control. Thus, detailed clinical information including clinical score was recorded for 152 of the 191 hips that were observed for 7 years; in 146 of these, a prosthesis of the first generation had been implanted. Furthermore, clinical scores were recorded in 75 of the 83 hips observed for 10 years since the femoral component had been changed in 2 hips due to periprosthetic fractures; in 6 other patients clinical scores were not recorded since they were followed up at other institutions. However, all complications and reoperations were registered at the 7- and 10-year follow-ups.

The radiological follow-up included investigation of the pelvis and the operated hips in all patients.

## Results

### Complications and reoperations

In the series of 191 hips that were followed for 7 years, an intraoperative fissure occurred in the trochanter region in 2 hips (1.0%). In both hips, the fissure healed after wiring without loosening of the custom stem. A partial lesion to the sciatic nerve occurred in 4 patients (2.1%) and to the anterior gluteal nerve in one hip (1.0%). All nerve lesions were verified by neurophysiological examination. Two of the sciatic nerve lesions recovered completely. Dislocation occurred in 3 hips (1.6%); all 3 became stable after closed reduction. No deep infections occurred. Deep venous thrombosis was diagnosed in 1.6%. 2 patients (1.0%) sustained a femoral fracture by a fall accident.

In the 83 hips that were followed for 10 years, one additional fracture occurred. Thus, the total rate of fractures within this group was 3.6%. There were no radiological signs of loosening in any of the fractures before the fracture occurred and bone ingrowth was found at the reoperations.

Reasons for, type of, and number of reoperations are given in Table 4. In both of the fractures that occurred before 7 years, stem replacement had to be performed because of extensive splitting of the periprosthetic bone. Since there were no revisions of the femoral stem other than those mentioned, the cumulative revision rate of the stems at 7 and 10 years was 1.0% and 2.4%, respectively. The fracture that occurred after 7 years was treated successfully with plate osteosynthesis. The liner had to be changed because of wear between 7 and 10 years postoperatively in 4 of 76 hips where a Duraloc acetabular component had been used. Two hips were explored because of pain, but without any positive findings.

**Table 4.** Reoperations

Reason for reoperation	Type of reoperation	7 years of follow-up (191 hips)	10 years of follow-up (83 hips)
Hip instability	Closed reduction	3	2
	Change of acetabular component	0	0
Femoral fracture	Osteosynthesis	0	1
	Change of femoral component	2	2
Polyethylene wear	Change of liner	0	4
Ectopic ossification	Removal	1	0
Pain	Surgical exploration	2	2

### Clinical scores

The mean values of Merle d'Aubigné preoperative pain score were equal in hips followed for 7 and 10 years (2.9). The same was true for the postoperative pain scores (5.7). The preoperative total scores improved from 9.6 to 17.2, and from 9.4 to 17.0, respectively. Thus, there was equal improvement in score values in the two observation groups. Thigh pain was noted at one or more follow-ups in 12% of 146 hips of the first generation, where a detailed registration of the clinical findings was performed over a 7-year period.

### Radiographic findings

No aseptic loosening was seen in hips observed for 7 and 10 years. Regarding the position of the implanted stems, they had usually ended in the planned position relative to the resection level of the femoral neck, or less than 3 mm higher. The maximum discrepancy was 5 mm. In very few cases, a slight varus or valgus position was seen.

### Clinical examples

**Figure 3.**
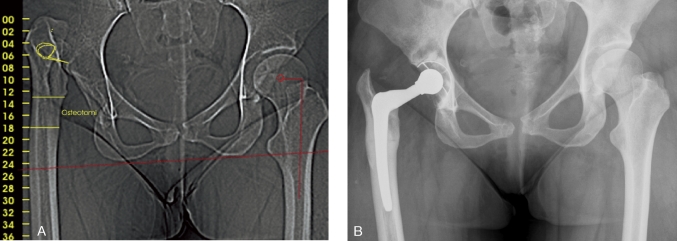
A. Scanogram of the right femur in a case of femoral head necrosis with high dislocation of the hip. B. Radiograph after subtrochanteric resection osteotomy and insertion of the custom stem. The osteotomy has healed.

**Figure 4.**
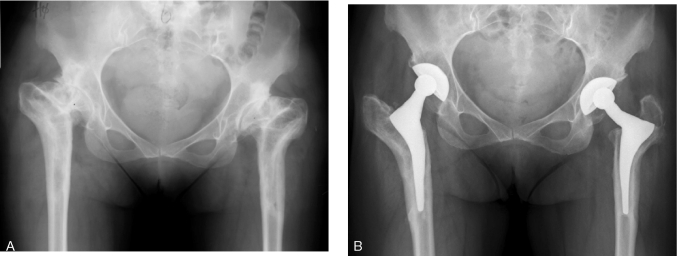
A. Bilateral hip dysplasia with valgus deformities after earlier subtrochanteric osteotomies. B. Radiograph after insertion of custom femoral stems bilaterally.

**Figure 5.**
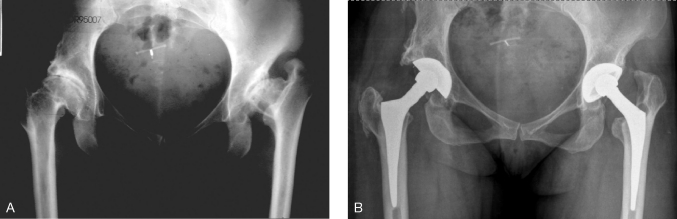
Preoperative (A) and postoperative (B) images in a case of bilateral CDH with osteoarthritis. Note that adequate medial femoral offset has been obtained despite the extremely narrow intramedullary cavities.

**Figure 6.**
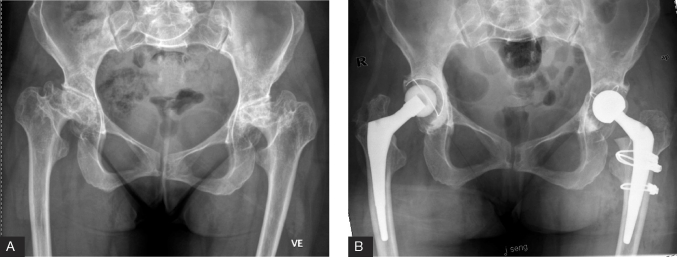
Preoperative (A) and postoperative (B) images of a case of dwarfism treated with custom stems. On the left side, an intraoperative fissure was treated with wires.

Some examples of what can be achieved by using custom stems are shown in Figures 2–6. Figure 2B demonstrates the results after insertion of the prosthesis that was planned as illustrated in Figure 1. The image demonstrates the suitability of a custom stem in congenital dysplasia with a low dislocation by making a prosthesis with a CCD-angle and neck length that give adequate medial femoral head offset and leg length (right hip). Figure 3 demonstrates a custom stem in a case of high dislocation of the hip. In this case, a shortening osteotomy of 2.5 cm was performed. The osteotomy was fixated with the prosthesis alone. The sections through the part of the femur to be removed were eliminated when the prosthesis was designed. Figure 4 demonstrates how a corrective osteotomy can be avoided by using a customized stem in severe deformities of the femur. Figure 5 shows that use of custom stems enables adequate medial femoral head offset despite there being an extremely narrow intramedullary canal. In such a case, it might be difficult to achieve normalization of offset by a standard stem since extremely narrow standard stems have a short neck. Finally, Figure 6 shows custom stems in dwarfism.

## Discussion

We have shown that the use of a custom uncemented stem has a low complication rate. Fissures in the trochanteric region occurred in 1.0% of the hips. In studies of uncemented standard stems, the incidence of this complication has varied between 1.2% and 8.8% (Petit 2004, Sanchez-Sotelo and Cabanela 2004, Toni et al. 2004, Videlain 2004, Lerch et al. 2007). It should be noted that the fissures that occurred in our series had no consequence for the final result. The low rate of femoral fractures (1.0% before 7 years) also shows that individually shaped stems can be used without increasing the risk of fracture.

Even after the slight downscaling of the largest stems, we occasionally experienced some difficulties in bringing the prosthesis down to the planned level without removing some compact cancellous bone. However, most of the stems have been adequately dimensioned and oversizing of a minority of the stems has not restrained the insertion of the stem or caused any other serious problems. We have abandoned the use of such a stem in only 3 out of more than 800 hips in which use of such a stem had been planned. In 1 hip, use of a custom stem had to be abandoned since it was impossible to reduce the joint because the planned lengthening had been too ambitious. Furthermore, one prosthesis could not be used since the stem was undersized. Finally, in 1 case the custom prosthesis could not be implanted because of a surgical error during the reaming of the severely deformed upper end of the femur.

The femoral fractures recorded in the groups of hips followed for either 7 or 10 years were all caused by fall injuries. There was no indication that oversize of the stem had contributed to these fractures. Moreover, the 1.0% rate of fractures in hips observed for 7 years and the 3.6% rate in hips followed for 10 years are not higher than figures reported after use of standard stems. Thus, Radl et al. (2000) found an incidence of 5.5% in 134 hips at an average of 5.5 years after insertion of proximally HA-coated stems. In cemented stems, Cook et al. (2008) found an incidence of fractures of 0.8% in 6,458 hips 5 years after operation and 3.5% at 10 years.

We have generally experienced that use of individually designed femoral stems is a reliable method to restore adequate medial femoral head offset. Furthermore, use of such components contributes to stability of the joint since a suitable femoral neck anteversion will be achieved. Thus, it is almost impossible to ream the cavity in a way that leads to a torsional malposition of the prosthesis. When the custom broach is brought down into the femoral canal, it is forced into the correct position by the compact endocortical bone. This is in contrast to what sometimes happens during broaching before insertion of a standard stem. A severely deformed femur might in some cases force a standard broach into a malrotated position, despite efforts made to avoid this. A considerable number of our patients had hips with a severely abnormal preoperative femoral anteversion due to congenital dysplasia. Despite this there was a relatively low dislocation rate, which may partly be due to the standardized femoral anteversion achieved with the custom technique. A dislocation rate of 1.6% after 7 years and 2.4% after 10 years is certainly low in series constituting a relatively high percentage of femurs with a highly increased preoperative anteversion. Also, an adequate reconstruction of the length of the femoral neck, which can be achieved by using individually designed femoral components, might contribute to good stability of the hip.

We have experienced that adequate correction of leg length discrepancy is easily achieved if the preoperative shortening is less than 3 cm. We have only experienced a deviation from the planned leg length of more than 5 mm in a few cases. If the leg length discrepancy is more than 3 cm, it is probably wise not to aim for a complete equalization of leg length due to the risk of nerve lesions.

In cases with a high dislocation of the hip, a prosthesis can be designed to stabilize the osteotomy after resection of a subtrochanteric segment. However, great care has to be taken to ensure that fixation with the prosthesis alone will be adequate. In some cases, additional plate fixation will be advisable. If necessary, the shortening osteotomy can be done distal to the prosthesis. In cases with low dislocation, a prosthesis with a moderately reduced CCD-angle can be designed to bring the head of the prosthesis down to the right anatomical level without doing a shortening osteotomy. In such cases, a careful stress analysis must be performed by the manufacturing company to ensure that such a design will not include a risk of fatigue fracture of the neck of the prosthesis.

Concerning the overall medium-term clinical results, the Merle d'Aubigné score at 7 years was excellent. However, despite the fact that the results were similar in the group observed for 10 years, it remains to be seen whether the long-term results will be maintained. It also remains to be seen whether the clinical results after operations with stems of the second generation will be different from the results presented where a first-generation stem was used in nearly all hips.

The incidence of thigh pain after treatment with the first-generation custom stems (12%) shows that this type of prosthesis has not completely eliminated the thigh pain phenomenon. Thigh pain markedly influenced the function in only 2 patients. In studies of uncemented standard stems with coatings for bony ingrowth, rates of thigh pain have been reported varying from 0% (Kim 2008) to close to 40% (Weidenhielm et al. 1995). In custom stems, Argenson et al. (2004) found less thigh pain in fully coated stems than in stems with only proximal coating. However, it is difficult to compare the incidence of thigh pain in various studies since the recording has not been standardized.Since no cases of aseptic loosening were recorded at the 7- and 10-year follow-up, it appears that an individual design contributes to safe fixation of the stem. Similar findings have been reported from other groups using custom stems (Wettstein et al. 2005, Leali et al. 2006, Akbar et al. 2009). During the past 10–15 years, however, the rate of aseptic loosening has also been substantially reduced in standard uncemented stems. According to a report from the Norwegian Arthroplasty Register (Hallan et al. 2007), all stems currently used in Norway perform excellently with survival of 96–100% at 10 years, with revision because of aseptic loosening as the endpoint. It can be argued that a registry study does not reveal aseptic loosening that has not led to revision surgery. However, there have been several reports indicating a total rate of aseptic loosening of between 0% and 5% after 10 years or more of observation (Emerson 2004, Moskal et al. 2004, Parvizi et al. 2004, Videlain 2004, Capello et al. 2006, Sharma and Brooks 2006, Yoon et al. 2008). Thus, it can hardly be argued that a customized stem should be taken into general use due to a safer fixation than that achieved with standard stems. However, it should be noticed that there was a considerable number of hips included in our study where a standard uncemented stem could not be used because of severe deformity of the upper femur. If an individually designed stem had not been available, a cemented stem would have had to be used in these hips—if necessary, combined with a corrective osteotomy.

A more favorable strain distribution to the proximal femur was found in in vitro studies when compared to a standard proximal coated stem (PCA stem) (Aamodt et al. 2001). It could therefore be expected that an individual design would contribute to more favorable bone remodeling. However, preliminary results of comparative DEXA studies have not shown less bone atrophy in the custom stems. This might be explained by the fact that the surgical trauma itself is a major reason for the bone loss, and that the strain distribution is modified by the ingrowth of bone. Results of these DEXA studies will be published later.

More liberal use of custom prostheses in hips with no major anatomical deformities, as seen during the last part of the present study period, might give cause for concern regarding cost since such prostheses are more expensive than standard stems. However, if their use is restricted to hips where there is a reasonable chance of improved results compared to what would be obtained using standard stems, the higher costs would be justified.

In summary, we have experienced that the use of custom uncemented stems is a reliable method to achieve adequate mechanics of the hip joint in terms of suitable medial femoral head offset and femoral neck anteversion, combined with optimal leg length correction. Furthermore, the individual fitting of the stems makes it possible to insert the stem into severely deformed femurs, reducing the need for corrective osteotomies. Use of such stems gives reliable fixation and excellent medium-term clinical results, but does not completely eliminate the risk of thigh pain following the use of uncemented stems. Custom stems are especially indicated in hips with abnormal size and geometry of the upper femur, but use of such stems might also be advantageous in hips without major deformities—particularly if the preoperative planning shows that it might be difficult to restore ideal biomechanics of the joint with a standard technique. The preoperative planning, like the operative technique, is easy, and the use of custom stems does not carry any additional risks compared to standard stems.
